# The Progeroid Phenotype of Ku80 Deficiency Is Dominant over DNA-PK_CS_ Deficiency

**DOI:** 10.1371/journal.pone.0093568

**Published:** 2014-04-16

**Authors:** Erwin Reiling, Martijn E. T. Dollé, Sameh A. Youssef, Moonsook Lee, Bhawani Nagarajah, Marianne Roodbergen, Piet de With, Alain de Bruin, Jan H. Hoeijmakers, Jan Vijg, Harry van Steeg, Paul Hasty

**Affiliations:** 1 National Institute for Public Health and the Environment, Bilthoven, The Netherlands; 2 Department of Cell Biology and Genetics, Center for Biomedical Genetics, Erasmus MC, Rotterdam, The Netherlands; 3 Faculty of Veterinary Medicine, Department of Pathobiology, Utrecht University, Utrecht, The Netherlands; 4 Department of Genetics, Albert Einstein College of Medicine, Bronx, New York, United States of America; 5 Department of Toxicogenetics, Leiden University Medical Center, Leiden, The Netherlands; 6 Department of Molecular Medicine and Institute of Biotechnology, Barshop Institute for Longevity and Aging Studies, Cancer Therapy and Research Center, University of Texas Health Science Center at San Antonio, San Antonio, Texas, United States of America; University of Pittsburgh, United States of America

## Abstract

Ku80 and DNA-PK_CS_ are both involved in the repair of double strand DNA breaks via the nonhomologous end joining (NHEJ) pathway. While *ku80^−/−^* mice exhibit a severely reduced lifespan and size, this phenotype is less pronounced in *dna-pk_cs_^−/−^* mice. However, these observations are based on independent studies with varying genetic backgrounds. Here, we generated *ku80^−/−^*, *dna-pk_cs_^−/−^* and double knock out mice in a C57Bl6/J*FVB F1 hybrid background and compared their lifespan, end of life pathology and mutation frequency in liver and spleen using a lacZ reporter. Our data confirm that inactivation of Ku80 and DNA-PK_CS_ causes reduced lifespan and bodyweights, which is most severe in *ku80^−/−^* mice. All mutant mice exhibited a strong increase in lymphoma incidence as well as other aging-related pathology (skin epidermal and adnexal atrophy, trabacular bone reduction, kidney tubular anisokaryosis, and cortical and medullar atrophy) and severe lymphoid depletion. LacZ mutation frequency analysis did not show strong differences in mutation frequencies between knock out and wild type mice. The *ku80^−/−^* mice had the most severe phenotype and the Ku80-mutation was dominant over the DNA-PK_CS_-mutation. Presumably, the more severe degenerative effect of Ku80 inactivation on lifespan compared to DNA-PK_CS_ inactivation is caused by additional functions of Ku80 or activity of free Ku70 since both Ku80 and DNA-PK_CS_ are essential for NHEJ.

## Introduction

DNA damage is known to be involved in tumorigenesis and aging. There are multiple DNA repair pathways that specialize in repairing a specific DNA lesion. One such pathway is nonhomologous end joining (NHEJ), which plays an import role in the repair of double strand breaks (DSBs). Ku80 and DNA-PK_CS_ are both components of the NHEJ pathway. Ku80 forms a heterodimer with Ku70, known as Ku, which binds to DNA ends at a DSB [1]. DNA-dependent protein kinase (DNA-PK) is a holo enzyme formed by a complex between Ku and DNA-depended protein kinase catalytic subunit (DNA-PK_CS_). DNA-PK_CS_, together with Artemis, Xrcc4-DNA ligase and Xrcc4-like factor, process DNA overhangs and ligation [2]–[4].

Cells with deletion of any of these NHEJ components show severe combined immunodeficiency (SCID) due to defects in assembling variable (diverse) joining (V(D)J) segments of antigens, genetic instability and hypersensitivity to DSB inducing agents [5]–[9]. However, there is some phenotypic variation. For instance, deletion of Ku70 and Ku80 results in reduced size, severely decreased lifespan, neuronal apoptosis and accelerated aging. Strikingly, in previous experiments using mixed backgrounds, these mice seem to be protected against tumor development, although they show a low level of thymic lymphomas [10], [11]. In contrast, such a phenotype is less pronounced in DNA-PK_CS_ knock out mice. Compared to the *ku70^−/−^* and *ku80^−/−^* mice, the early aging phenotype for the *dna-pk_cs_^−/−^* mice is less severe and best observed with shortened telomeres [12]–[14]. Yet, these studies were performed in different genetic backgrounds in different labs. Therefore, phenotypic differences could be the result of inconsistent genetic backgrounds and environments. On the other hand, some phenotype differences can be explained by additional functions of the deleted NHEJ components or that a non-deleted NHEJ component is deleterious in absence of the deleted protein. Such deleterious effects have been observed previously [15], [16]. For instance, DNA ligase IV deficiency is lethal in presence of Ku80 but lethality is rescued by deletion of Ku80 [16]. Furthermore, the prenatal lethal phenotype of Xrcc4 deficient mice is partly rescued by p53 knock out [15], that negates neuronal apoptosis.

Here, we generated cohorts of *ku80^−/−^*, *dna-pk_cs_^−/−^*, double knockout and wild type mice in an identical genetic background and compared lifespan, development of body weight, end-of-life pathology and accumulation of genetic insults (LacZ mutant frequencies in liver and spleen). We find that *ku80^−/−^* mice exhibit a more sever phenotype than *dna-pk_cs_^−/−^* mice while the double mutant mice are as severe as the *ku80^−/−^* mice. These observations suggest that Ku80 has additional functions that do not require DNA-PK_CS_ or that free Ku70 has a deleterious affect in the absence of Ku80. Both possibilities are in line with our previous reports that show Ku70 and Ku80 have functions independent of the Ku heterodimer [17].

## Results

**Figure 1:**
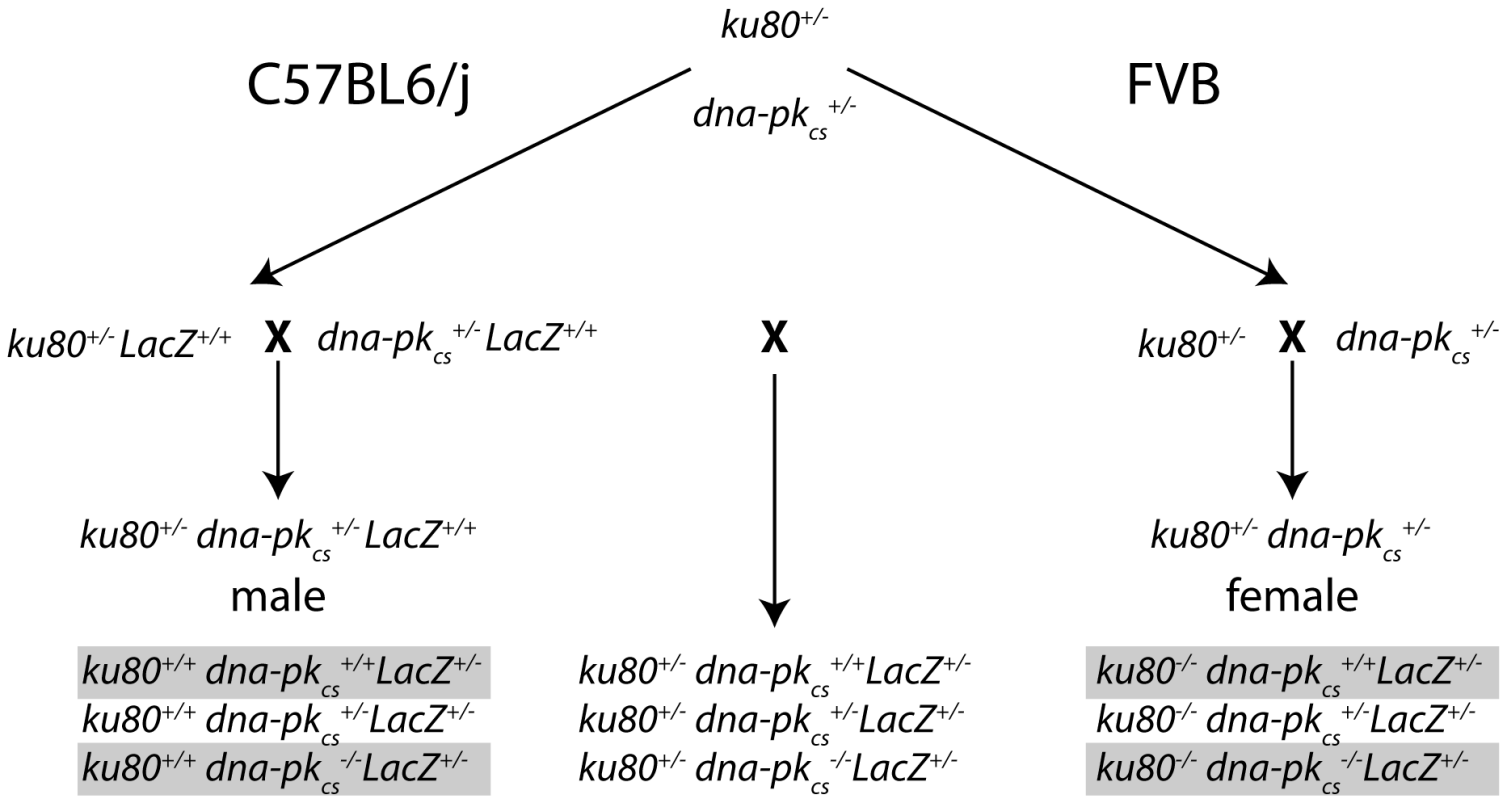
Breeding strategy to generate the knockout cohorts. *Ku80^+/−^* and *dna-pk_cs_* ^+/−^ animals are backcrossed for multiple generations to C57Bl6/J-pUR288 (left of dashed line) and FVB (right of dashed line) background. Double heterozygous knock out C57BL6/J male mice are crossed with double heterozygous knock out FVB female mice. Using this strategy all four desired cohorts are generated as F1 hybrids (grey boxes).

Previous reports suggest that *ku80^−/−^* mutant mice exhibit a more severe phenotype than *dna-pk_CS_^−/−^* mice. This was not predicted since both Ku80 and DNA-PK_CS_ are essential for NHEJ. Thus, the phenotypic differences could be due to divergent function or divergent genetic background and environment. Alternatively, one of the components could be toxic in the absence of the deleted protein. To better understand the reason for these different phenotypes we generated *ku80^−/−^* and *dna-pk_cs_^−/−^* and double mutant cohorts in the same genetic background using double heterozygous breeders (*ku80^+/−^ dna-pk_CS_^+/−^*) as shown in figure 1. The breeding pairs were composed of C57Bl6/J-pUR288 males and FVB females so the cohorts were F1 brothers and sisters raised in the same cages. Thus, all cohorts are controlled for genetic background and environment so any phenotypic difference between the *ku80^−/−^* and *dna-pk_cs_^−/−^* cohorts must be due to divergent function. The double mutant mice will also help determine if one component assumes a toxic activity after the other is deleted.

**Figure 2:**
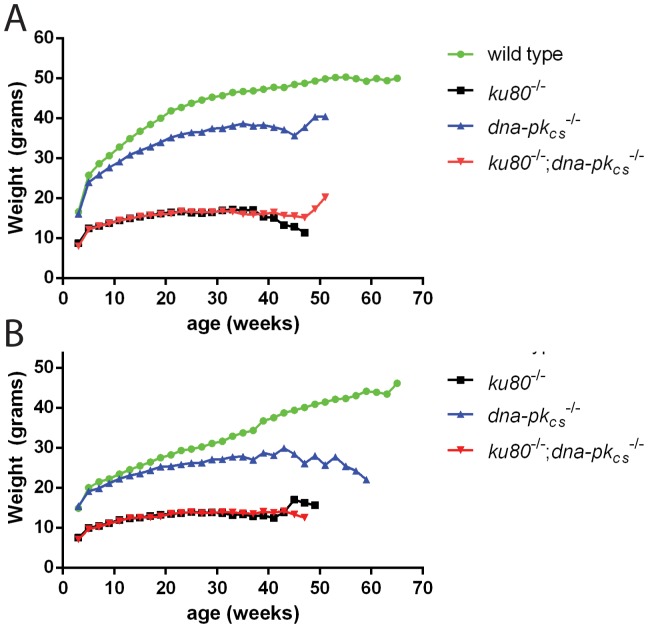
Mean body weights. (A) Males. (B) Females.

During the longevity study, all mice were weighed every two weeks. As can be seen in figure 2, all mutant mice were smaller compared to wild type mice. *ku80^−/−^* and double knock out mice weighted between 10 and 20 grams, while *dna-pk_cs_^−/−^* mice weighted between 15 and 35 grams and were therefore considerably heavier than *ku80^−/−^* and double knock out mice. Due to differences in body size, organ weights differed significantly between the different genotypes (Figure S1). However, after correction for bodyweight, most organ weights were comparable. The exception is the testis, which was significantly heavier in *dna-pk_cs_^−/−^* mice compared to the other knock out mice. Marginal significant differences were observed for males in relative organ weight for kidney, liver, spleen and heart. The wild type cohort was terminated after the final mutant mouse died since their life span appeared much longer; therefore, no end of life organ weight data were collected for this cohort. Thus, deletion of either Ku80 or DNA-PK_CS_ reduced body weight with Ku80-deletion being more severe and dominant to DNA-PK_CS_ -deletion.

**Figure 3:**
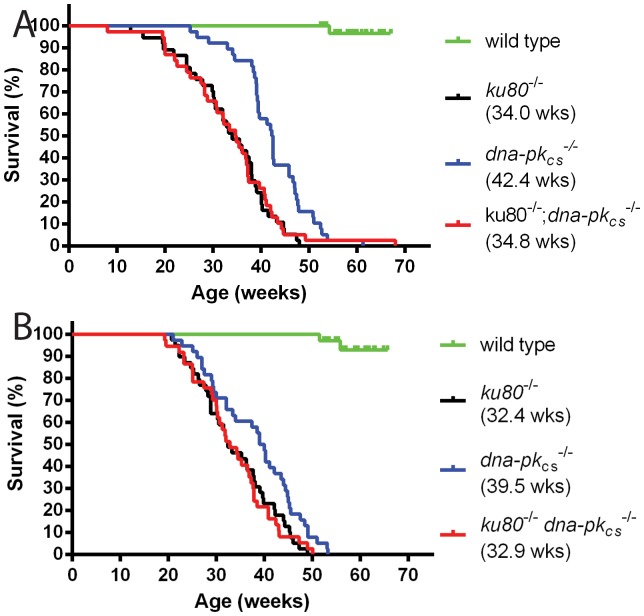
Survival curves. Median survivals are shown in the figure legends in parentheses. *dna-pk_cs_^−/−^* mice live significantly longer compared to *ku80^−/−^* mice in both male (*p* = 0.01) and females (*p* = 8.7*10^−6^) mice. Survival of double knock out mice is identical to *ku80^−/−^*. (A) Males. (B) Females.

All mutant mice displayed a severely reduced lifespan compared to wild type mice (Fig. 3). Previously, we showed that *dna-pk_cs_^−/−^* mice were loner-lived than *ku80^−/−^* mice for both males and females (*p* = 0.01 and *p* = 8.7*10^−6^ respectively) [17]. Here we also show that the double knock out mice had the same short life span as *ku80^−/−^* mice (Fig. 3). Thus, deletion of either Ku80 or DNA-PK_CS_ reduced life span with Ku80-deletion being more severe and dominant to DNA-PK_CS_ -deletion.

We screened for aging pathology in a selected set of animals (n = 4–5). Results are shown in table 1. Evidence of aging was found in the kidney. Mild increased renal tubular anisokaryosis was observed in all knock out models. Moderate to severe renal tubulonephrosis was present in all mutant cohorts compared to wild type animals. Analysis of skin revealed moderate to severe epidermal and adnexal atrophy. Dermis and epidermis thickness was reduced in all mutant mice. Finally, all mutant mice showed mild to moderate reduction in trabecular bone thickness, which is indicative of osteopenia. There were no differences between genotypes in anisokaryosis or lipofuscin accumulation in liver, which was previously observed in aged wild type mice [18]. Thus, deletion of either Ku80 or DNA-PK_CS_ caused an early onset of many but not all aging characteristics previously reported for wild type mice.

**Table 1:** Histopathology non-neoplastic lesions.

Lesion	Median pathology score (min – max)							
	*ku80* ^−/−^		*dna-pk_cs_^−/−^*		*k*-/-;*d*-/-		Wild type	
	Male (41w)	Female (42w)	Male (45w)	Female (45w)	Male (42w)	Female (40w)	Male (37w)	Female (40w)
Liver anisokaryosis	2 (1-3)	1.5 (1-3)	3 (1-3)	2* (2-3)	2 (2-3)	2.5 (1-3)	1 (1-2)	1 (1-2)
Liver lipofuscin	1 (1-1)	1 (1-1)	1 (1-1)	1 (1-2)	1 (1-1)	1 (1-1)	1 (1-1)	1 (1-1)
Renal tubular anisokaryosis	2 (1-3)	2 (1-3)*	2 (1-3)	2 (2-3)	3* (2-3)	2 (1-3)	1 (1-2)	1 (1-1)
Renal tubulonephrosis	3*** (1-3)	3*** (2-4)	3*** (1-3)	4*** (2-4)	2*** (1-3)	3*** (1-3)	0 (0-0)	0 (0-0)
Epidermal atrophy	2** (1-3)	2* (1-3)	3** (2-4)	3* (2-3)	3** (1-4)	3* (2-3)	0 (0-0)	0 (0-0)
Adnexal atrophy	1** (1-2)	1* (0-1)	2.5** (1-4)	3** (1-3)	2* (0-4)	2** (1-3)	0 (0-0)	0 (0-0)
Lymphoid depletion spleen	4* (3-4)	4* (3-5)	3* (3-3)	3* (3-3)	4* (3-5)	3** (2-4)	0 (0-0)	0 (0-0)
Lymphoid depletion mes ln	4* (4-5)	3.5** (2-5)	2* (1-2)	1* (1-2)	4** (4-4)	4** (4-4)	0 (0-0)	0 (0-0)
**Tissue thickness in µm (St. dev.)**								
Dermis and epidermis	253.8* (56.9)	151.3* (11.7)	222.0** (38.3)	172.8 (58.7)	252.0* (99.0)	159.0 (86.3)	374.3 (50.6)	247.3 (43.4)
Trabecular bone	130.8* (36.7)	131.0* (38.2)	124.5* (18.8)	139.8* (25.5)	138.4 (43.1)	118.8** (10.6)	184.0 (19.4)	185.3 (22.4)

4–5 animals examined per group. All selected mutant mice are at end of life, wild type mice are aged matched to mutant mice.

Mean age in weeks is shown (w).

mes ln: mesenterial lymph node.

*k*-/-;*d*-/-: *ku80* ^−/−^; *dna-pk_cs_^−/−^*.

St. Dev.: Standard deviation.

*p*-values based on comparison with wild type animals.

* *p*<0.05.

** *p*<0.01.

*** *p*<0.001.

Mutant cohorts showed a high tumor incidence at the time of death compared to age-matched controls (table 2). Most tumors were observed in the thymus followed by liver tumors. Pathological examination showed that tumors were almost exclusively lymphomas. CD3 staining showed lymphomas to be of t-cell origin (three mice analyzed for each genotype). There were also rare hepatic adenomas (n = 6), a hepatic carcinoma (n = 1) and a bronchiolo-alveolar carcinoma (n = 1), which might be caused by the C57Bl6/J background according to the Mouse Tumor Database (http://tumor.informatics.jax.org/mtbwi/index.do). Lymphomas were also present in other organs than thymus with a high frequency in liver, kidney, spleen and lymph nodes. Lymphoid tissues of mutant animals, not affected by lymphomas, showed severe lymphoid depletion (table 1) as expected due to the failure to complete V(D)J recombination. This was observed in spleen and mesenterial lymph nodes and was severe in most mutant animals, with exception of female *dna-pk_cs_^−/−^* mice, which showed a mild to moderate lymphoid depletion in mesenterial lymph nodes. Animals affected by lymphoid depletion also showed an increased myeloid/erythroid ratio. Although tumor incidence was slightly higher in *ku80^−/−^* mice compared to *dna-pk_cs_^−/−^* and the highest in double knock out mice, this did not reach statistical significance. No synergistic effects of DNA-PK_CS_ deletion in addition to Ku80 deletion were found nor did deletion of one protein ameliorate the phenotype for deletion of the other protein.

**Table 2:** Histopathology neoplastic lesions.

Organ	Tumor	*ku80* ^−/−^		*dna-pk_cs_^−/−^*		*k*-/-;*d*-/-	
		Male (n = 25)	Female (n = 15)	Male (n = 27)	Female (n = 10)	Male (n = 24)	Female (n = 21)
Liver	lymphoma	9	5	4	7	5	2
	adenoma	1	1			3	1
	carcinoma					1	
Lung	lymphoma	1					
	carcinoma		1				
Pancreas	lymphoma					1	
Kidney	lymphoma	5	2	3	6	4	5
Thymus	lymphoma*	23	14	21	9	19	15
ovary/uterus	lymphoma		1		2		
Spleen	lymphoma	4		2	3	3	
mes. lm. nd.	lymphoma	3	2	5	5	3	
ax. lm. nd.	lymphoma	3	3	5	5	5	
Skin	lymphoma				1		
Testis	lymphoma	1					
bone marrow**	lymphoma	2	1	2	2	1	1
pituitary gland	lymphoma				1		
Tumor incidence (%)		67	39	55	24	75	51

Mentioned n-numbers are tumor bearing animals available for microscopical examination.

*k*-/-;*d*-/-: *ku80*-/-; *dna-pk_cs_^−/−^*.

mes. lm. nd.: mesenterial lymph node.

ax. lm. nd.: axillary lymph node.

*∼5 thymus tumor preparations were microscopically examined per group and found to be all lymphomas. All other macroscopic neoplastic lesions observed in thymus are assumed to be lymphomas as well.

** analyzed in femur.

Tumor incidence based on all animals from longevity cohort, including those not microscopically examined.

**Figure 4:**
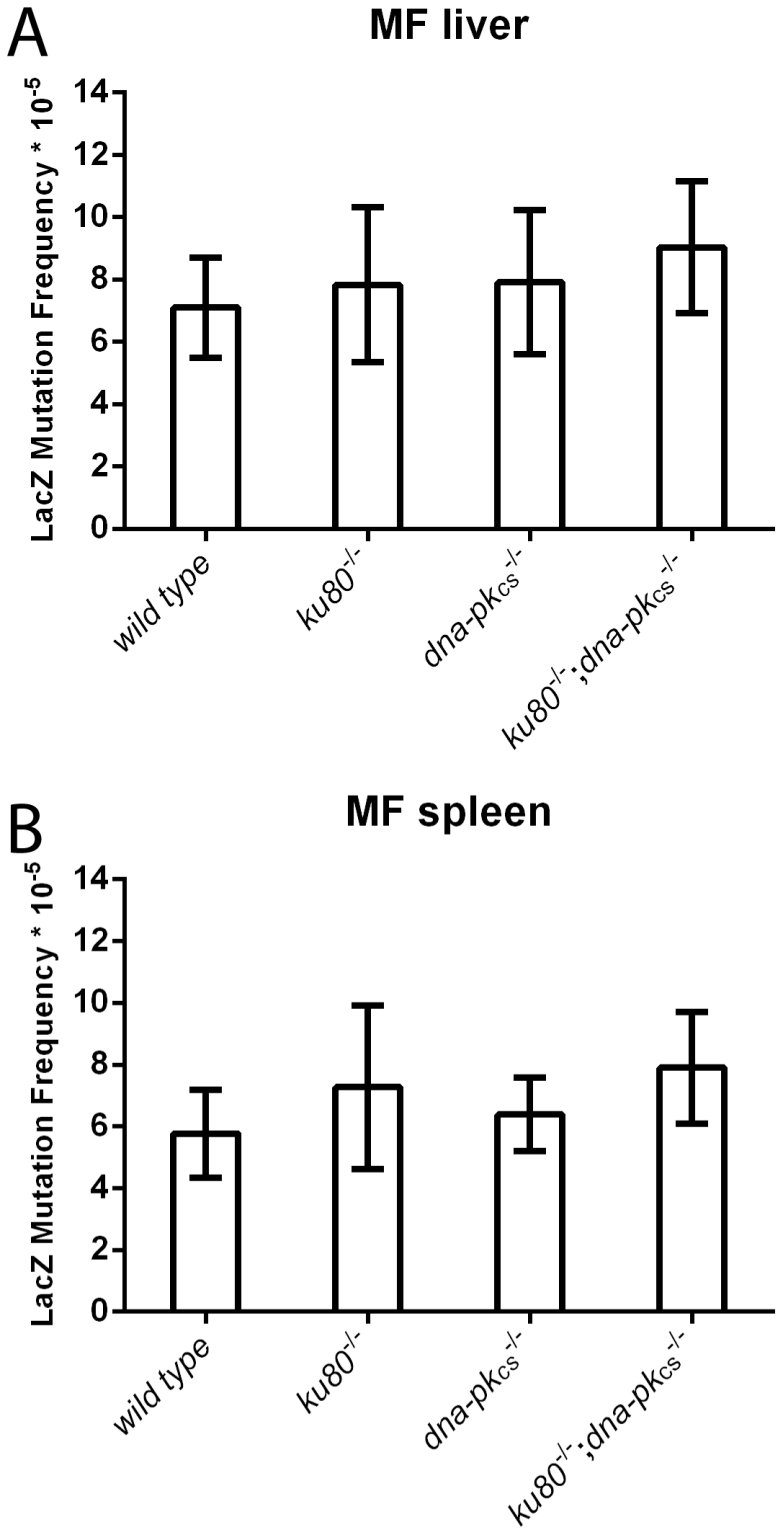
LacZ mutant frequency. Error bars represent standard deviation. (A) Mutant frequency in liver. (B) Mutant frequency in spleen.

Since Ku80 and DNA-PK_CS_ are DNA damage repair proteins, it would be expected that deletion of these proteins affect mutant frequency. In order to test the LacZ mutant frequencies in liver and spleen, 5 *ku80^−/−^*, *dna-pk_cs_^−/−^* and double knock males were killed at 38.7, 41.3 and 33.7 weeks, which corresponds approximately with their median lifespan (figure 3). Because the wild type cohort was not maintained beyond median age, we selected the 5 oldest wild type males available at the same observation time (mean age is 37.3 weeks). No significant differences in mutant frequency were observed between genotypes in both liver as spleen (figure 4). We did observe a trend towards increased mutant frequency in double knock out compared to wild type mice in spleen, but this did not reach statistical significance (*p* = 0.07).

## Discussion

Previously, it was reported that Ku80 inactivation resulted in reduced cancer and accelerated aging [12], [14], [19] while a similar but less pronounced phenotype was observed with DNA-PK_CS_ inactivation [12]–[14]. This phenotypic variance is unexpected since both Ku80 and DNA-PK_CS_ are essential for NHEJ. Therefore, these different phenotypes could be due to differences in genetic background and/or environment. However, our experiment presented here does not support either possibility since we controlled for both genetic background and environment but still observed a disparity in phenotypes. Alternatively, *ku80^−/−^* mice could exhibit a more severe phenotype than *dna-pk_CS_^−/−^* mice, if DNA-PK_CS_ is toxic in the absence of Ku80. There is president for this possibility since Ku80-deletion rescued embryonic lethality for *DNA ligase IV*-null mice showing that Ku80 was toxic in the absence of DNA ligase IV. However, deleting DNA-PK_CS_ did not ameliorate the Ku80-mutant phenotype. Instead the double-mutant mice exhibited the same severe phenotype as the Ku80-mutant mice. Therefore, our experiment suggests that Ku80 has extra-NHEJ function or that free Ku70 has a toxic activity in the absence of Ku80.

Our previously published data supports both possibilities. First we found that Ku80 has extra-NHEJ activity in mice by comparing p53-mutant mice deleted for Ku80 or Ku70 or both [20]. For this experiment, we found that *ku70^−/−^ p53^−/−^* mice lived longer than *ku80^−/−^ p53^−/−^* mice. This life span extension required Ku80 since the triple-mutant mice had the same life span as the *ku80^−/−^ p53^−/−^* mice. The *ku70^−/−^ p53^−/−^* mice lived longer because they had a lower incidence of pro-B cell lymphoma that was restored with deletion of Ku80. Therefore, these experiments suggest that Ku80 has extra-NHEJ activity. In addition, we showed that free Ku70 and free Ku80 bind to apurinic/apyrimidinic (AP) sites and that free Ku70 inhibits AP endonuclease 1 [17]. Another group also showed that Ku and DNA-PK_CS_ inhibited AP site cleavage by APE1 [21]. Thus, Ku80 and Ku70 have activity that is separable from the Ku heterodimer and NHEJ that could exacerbate the Ku80-mutant phenotype and account for the phenotypic disparity between deleting Ku80 and DNA-PK_CS_.

Ku80 as well as DNA-PK_CS_ inactivation accelerated aging. These characteristics included skin atrophy, femur osteopenia, renal tubular anisokaryosis and cortical and medullar atrophy. Accelerated aging was observed previously in Ku80 and DNA-PK_CS_ negative mice in different genetic backgrounds and environments suggesting that early aging is not sensitive to genetic and environmental changes [10], [22] [19].

Deletion of either Ku80 or DNA-PK_CS_ also increased cancer risk. Pathological examination revealed that tumors were predominantly lymphomas. Most lymphomas were observed in the thymus. Lymphomas in non-lymphoid tissues (e.g. liver and lung) are presumably metastases.

By contrast to this report, our prior reports show that *ku80^−/−^* and *ku70^−/−^* mice exhibited low levels of cancer [10], [11]. Furthermore, deleting Ku80 in a familial adenomatous polyposis mouse model (APC^MIN^) reduced intestinal tumors and increased life span [23]. We proposed that Ku80-deletion reduced cancer levels due to constitutive activation of the p53 DNA damage response and possibly other responses [24]. In support, deleting p53 greatly enhanced pro-B cell lymphomas and medulloblastoma in *ku80^−/−^* and *ku70^−/−^* mice [20], [25], [26]. Thus, difference in cancer incidences between our prior reports and this report suggest genetic background influence cancer incidence in the *ku80^−/−^* mice. This is possible since the former reports described mice derived from a 129*C57Bl6/J hybrid background while the current report describes mice derived from a C57Bl6/J*FVB F1 hybrid background.

Furthermore, we observed an increased myeloid/erythroid ratio that suggests true myeloid hyperplasia. However, complete blood counts are needed to confirm this. By contrast, we observed severe lymphoid depletion in all knock out cohorts. Lymphoid depletion is a known phenotype in SCID mice, which have a defective V(D)J recombination [27], [28]. Since *ku80^−/−^* and *dna-pk_cs_^−/−^* are SCID [9], [22], it is not surprising that lymphoid depletion was found in the knock out cohorts. Therefore, this element of the phenotype is not age-related. The increased myeloid/erythroid ratio could be the result of a compensatory response to this lymphoid depletion, triggering myeloid hyperplasia and lymphoma development.

Since Ku80 and DNA-PK_CS_ mutant mice have a defective DSB repair system, one expects accumulation of DNA damage. However, LacZ mutant frequency analysis could not confirm the previously observed increased mutant frequency in spleen [29]. Although our data do show a weak trend into the same direction, variation is too large and effect sizes too small to achieve statistical significance. Previously we had to separate the small mutations from the big mutations before a difference could be seen since the former was reduced while the latter was increased.

In conclusion, we have shown that deletion of the NHEJ components Ku80 and DNA-PK_CS_ in a C57Bl6/J*FVB F1 hybrid background resulted in a accelerated aging phenotype and a strongly increased incidence of lymphomas and that simultaneous deletion does not further enhance these characteristics. Yet, Ku80-deletion was more severe than DNA-PK_CS_ –deletion. This observation is consistent with the possibility that Ku80 has an extra-NHEJ function or that free Ku70 is toxic in the absence of Ku80.

## Materials and Methods

### Ethics Statement

All animal work was approved by the ethics committee of the National Institutes for Public Health and the Environment (RIVM), Antonie van Leeuwenhoeklaan, Bilthoven, The Netherlands, IACUC protocol #:99047x.

### Mice breeding

Ku80 null mice are not viable in a pure C57BL6/J background [30]. Therefore, we generated F1 hybrid animals with a C57Bl6/J*FVB background. This resulted in viable offspring and prevented C57BL6/J related ulcerative dermatitis [31]. *ku80^−/−^* mice [9] and *dna-pk_cs_^−/−^* mice [32] were imported in our SPF facility, rederived and back crossed to C57Bl6/JIco (Charles River, France) and FVB/NHanHsd (Harlan, Germany) using a speed congenics approach [33]. Knock out mice in C57Bl6/JIco background were maintained by backcrossing with C57Bl6/J-pUR288 (LacZ locus integrated at chromosome 13 [34]) generating heterozygous knock out animals which are homozygous for pUR288. Knock out mice on FVB background were maintained by backcrossing with pure FVB wild type animals. Cohorts with single and double knock out animals were generated using double heterozygous knock out breeders (*ku80* ^+/−^; *dna-pk_cs_* ^+/−^). All male breeders were on C57Bl6/J-pUR288 and all female breeders on FVB background. Using this breeding strategy, all genotypes could be generated from the same breeding colony and all experimental mice were C57Bl6/J*FVB F1 hybrids carrying one LacZ allele. The breeding scheme is depicted in figure 1.

This study was carried out in strict accordance with institutional guidelines and regulations. All animal work was approved by the ethics committee of the National Institutes for Public Health and the Environment (RIVM), Antonie van Leeuwenhoeklaan, Bilthoven, The Netherlands, IACUC protocol #:99047x. These were survival studies; therefore, mice were monitored every day without intervention. Moribund mice were sacrificed with ketamine/xylazine anesthesia followed by cervical dislocation and all efforts were made to minimize suffering and discomfort. Criteria for moribund were >15% weight loss within 2 weeks, not responsive to touch, prominent appearance of ribs, spine and hips, hunch body position, matted fur, or a visible tumor.

### Study setup

Each cohort consisted of 45 males and 45 females. All animals were maintained under specific pathogen free conditions and were fed ad libitum using CRM pelleted maintenance diet (Special Diet Services, UK). A 12 hr/12 hr dark/light cycle was maintained and temperature was 20°C. Animals were maintained until death or moribund. Moribund animals were euthanized by exsanguinations and major organs were isolated and partly stored in formaldehyde and partly snap frozen in liquid nitrogen. Results from *ku80^−/−^* and *dna-pk_cs_^−/−^* longevity cohorts were also used in a study comparing Ku70 and Ku80 function [17]. In addition, 5 male animals of all genotypes were euthanized at approximately median lifespan for LacZ mutant frequency analysis.

### Pathology

Pathology lesions in mutant animals were compared with those in age matched wild type littermates. Representative sections from the liver, kidney, thymus, spleen, axillary and mesenteric lymph nodes, femur, vertebra, uterus, ovaries, testes, and accessory male genital glands were processed (n = 4–5 per genotype), stained with Hematoxylin and Eosin, and microscopically examined for the presence of histopathologic lesions. All tumors macroscopically identified during necropsy were also prepared for histopathologic examination Severity score of all recorded lesions was semi-quantitatively assessed. Scores were given as absent (0), subtle (1), mild (2), moderate (3), severe (4), and massive (5) for each criteria. Digital images from the femur cortical bone at mid-shaft area, and skin were taken for morphometric analysis using Labsense image analysis software (Olympus). The thickness of femur cortical bone thickness, and skin thickness (dermis and epidermis with exclusion of subcutaneous fat) were measured using arbitrary line option.

### CD3 staining

After deparaffinization, rehydration and citrate buffer pretreatment, sections were incubated with rabbit polyclonal antibody to the human CD3 molecule (DAKO Corporation- Code-Nr. A 0452), diluted 1∶250 in 10% normal goat serum, for 30 minutes at room temperature. The remainder of the procedure was accomplished according to the manufacturer's instructions. Normal goat serum was substituted for the primary antibody as a negative control. Mouse lymphoid tissue was used as a positive control.

### LacZ mutant frequency analysis

LacZ mutant frequency was assessed in liver and spleen using the pUR288(lacZ) reporter. This assay has been described previously [34]. In short, total DNA was extracted from liver and spleen using a phenol/chloroform/iso-amyl alcohol extraction (25∶24∶1) and digested with the endonuclease HindIII in the presence of magnetic beads, coated with lacI-lacZ fusion protein. After digestion, magnetic beads were washed and DNA fragments eluted using isopropyl-L-thio-B-D-galactopyranoside. DNA fragments were circularized with T4 DNA ligase and transformed into *E. Coli* (ΔlacZ, galE-) bacteria. Of the transformed bacteria, one thousandth was plated on x-gal plates and the remainder on selective p-gal plates. The mutant frequency was defined as the amount of colonies on the selective plate divided by the amount of colonies on the X-gal plate multiplied by 1000 (dilution factor).

### Statistical analysis

Survival curves were analyzed using a log rank test (Graphpad Prism 5.04, GraphPad Software Inc, La Jolla, CA, USA). Pathological scores were compared using a two-tailed Mann-Whitney U test and mutant frequencies were compared using a T-Test (IBM SPSS Statistics 19, SPSS Inc - IBM, New York, USA).

## Supporting Information

Figure S1 a. Relative organ weights in males. b. Relative organ weights in females. c. Absolute organ weights in males. d. Absolute organ weights in females.
(DOC)
