# Supplementary material for: The Progeroid Phenotype of Ku80 Deficiency Is Dominant over DNA-PKCS Deficiency
Source: PLoS One. 2014 Apr 16;9(4):e93568. doi: 10.1371/journal.pone.0093568 (PMC3989187; doi:10.1371/journal.pone.0093568)
Supplement: Figure S1 — a. Relative organ weights in males. b. Relative organ weights in females. c. Absolute organ weights in males. d. Absolute organ weights in females. (DOC) [file pone.0093568.s001.doc]

Supplementary figure 1a. Relative organ weights in males

******

******

*****

*****

*****

*****

*****

*****

No end of life data of wild type animals available because wild type cohorts were terminated

* *p*<0.05

** *p*<0.001Supplementary figure 1b. Relative organ weights in females

No end of life data of wild type animals available because wild type cohorts were terminated

No significant differences in relative organ weights in females were observed.

Supplementary figure 1c. Absolute organ weights in males

******

******

******

******

******

******

******

******

******

******

No end of life data of wild type animals available because wild type cohorts were terminated

***p*<0.001

Supplementary figure 1d. Absolute organ weights in females

******

******

*****

*****

*****

******

******

*****

*****

No end of life data of wild type animals available because wild type cohorts were terminated

* *p*<0.05

** *p*<0.001
